# Nrf2 regulates gene-environment interactions in an animal model of intrauterine inflammation: Implications for preterm birth and prematurity

**DOI:** 10.1038/srep40194

**Published:** 2017-01-10

**Authors:** Thomas E. Sussan, Kuladeep Sudini, C. Conover Talbot, Xiaobin Wang, Marsha Wills-Karp, Irina Burd, Shyam Biswal

**Affiliations:** 1 Department of Environmental Health and Engineering, Johns Hopkins Bloomberg School of Public Health, Baltimore, MD, USA; 2 Institute for Basic Biomedical Sciences, Johns Hopkins University School of Medicine, Baltimore, MD, USA; 3 Center on the Early Life Origins of Disease, Department of Population, Family and Reproductive Health, Johns Hopkins Bloomberg School of Public Health, Baltimore, MD, USA; 4 Integrated Research Center for Fetal Medicine, Department of Gynecology and Obstetrics, Johns Hopkins University School of Medicine, Baltimore, MD, USA

## Abstract

Preterm birth (PTB) is the leading cause of neonatal mortality, and surviving infants are at increased risk for lifelong disabilities. Intrauterine inflammation is an etiological factor that drives PTB, and oxidative stress is associated with PTB. Nuclear erythroid 2-related factor 2 (*Nrf2*) is a redox-sensitive transcription factor that is the key regulator of the response to oxidative and inflammatory stress. Here, we used the established mouse model of intrauterine inflammation-induced PTB to determine whether Nrf2 is a modifier of susceptibility to PTB and prematurity-related morbidity and mortality in the offspring. We determined that Nr2-deficient (Nrf2^−/−^) mice exhibited a greater sensitivity to intrauterine inflammation, as indicated by decreased time to delivery, reduced birthweight, and 100% mortality. Placentas from preterm Nrf2^−/−^ mice showed elevated levels of markers of inflammation, oxidative stress, and cell death, and transcriptomic analysis identified numerous key signaling pathways that were differentially expressed between wild-type (WT) and Nrf2^−/−^ mice in both preterm and control samples. Thus, Nrf2 could be a critical factor for gene-environment interactions that may determine susceptibility to PTB. Further studies are needed to determine if Nrf2 is a viable therapeutic target in women who are at risk for PTB and associated complications in the affected offspring.

Preterm birth (PTB) is the leading cause of neonatal morbidity and mortality worldwide, and it represents a major challenge to public health. There are approximately 15 million PTBs per year worldwide, which are responsible for 1 million deaths^1^. In the US, 1 in 9 births are preterm, which is the leading cause of infant mortality. The rate of PTBs in the US has increased by as much as 30% during the last 25 years^2^. Although the survival rate of newborns born preterm has increased dramatically over the past several decades^3^, these surviving preterm newborns are at increased risk for numerous short-term and long-term health disparities, thus representing an early life origin of adult disease. Preterm newborns have altered lung development, immature immune systems, and elevated oxidative stress, which predispose them to a variety of diseases later in life, including cognitive/behavioral deficits, reduced growth, blindness, deafness, gastrointestinal issues, chronic respiratory diseases^4^,^5^,^6^,^7^,^8^, and adverse metabolic outcome^9^. Thus, prevention of preterm delivery has important implications for health across the lifespan.

Susceptibility to PTB is determined by a combination of genetic and environmental factors. Environmental exposures that induce oxidative stress and inflammation are often associated with PTB, while genetic variants that alter the response to oxidative stress and inflammation contribute to this risk^10^,^11^. However our knowledge of gene-environment interactions that determine susceptibility to PTB and complications associated with exposure to intrauterine inflammation remains limited, and an improved mechanistic understanding of these interactions would help in developing effective preventive strategies.

Histologic and microbiological findings indicate that focal infection and inflammation play a significant role in the premature rupture of the fetal membranes and the pathogenesis of PTB. Preterm neonates and mothers have elevated levels of markers of oxidative stress, which negatively correlate with birthweight and gestational age at birth^12^,^13^,^14^,^15^,^16^,^17^. Elevated levels of markers of oxidative stress have been detected in preterm infants beyond their first 100 days of life^18^, suggesting that oxidative stress established prenatally may persist throughout infancy. Additionally, peripheral blood monocytes from preterm newborns exhibit impaired DNA repair capacity, suggesting that preterm infants have a defect in their ability to detoxify oxidative stress^19^. Collectively, there is compelling evidence that maternal and neonatal inflammation and oxidative stress are primary etiological determinants of PTB, which may persist for several months, leading to long-term adverse outcomes.

The transcription factor *nuclear factor (erythroid-derived 2)-like 2 (Nrf2)* is a key regulator of numerous cytoprotective proteins, including antioxidants, xenobiotic detoxification enzymes, and proteasomal pathway proteins^20^,^21^. The primary function of Nrf2 is to regulate basal antioxidant capacity and to respond to changes in the oxidative environment to initiate an adaptive transcriptional response to counteract environmental stress and to protect from the resulting cellular damage. Nrf2 is a pleiotropic regulator of numerous pathways, although the inducible targets of Nrf2 are primarily categorized as antioxidative genes. The Nrf2-dependent antioxidative response utilizes multiple pathways, such as (*a*) providing direct antioxidants, (*b*) encoding enzymes that directly inactivate oxidants, (*c*) increasing levels of glutathione and thioredoxin synthesis and regeneration, (*d*) stimulating NADPH synthesis, (*e*) enhancing toxin export via the multidrug response transporters, (*f*) inhibiting cytokine-mediated inflammation, (*g*) enhancing recognition, repair, and removal of damaged proteins, and (*h*) increasing chaperones and regulating post-translational modifications^22^. Thus, Nrf2 is a prolific and ubiquitous regulator of multiple pathways that counteract oxidative stress and inflammation.

We and others have previously demonstrated that Nrf2 is the key modifier of oxidative stress and inflammation in numerous disease models, and Nrf2 can be targeted pharmacologically for therapeutic intervention^23^,^24^,^25^,^26^,^27^,^28^,^29^. Previously, we validated a mouse model of intrauterine inflammation, in which intrauterine lipopolysaccharide (LPS) induces inflammation, oxidative stress, and PTB^30^,^31^. Thus, we hypothesized that Nrf2 deficiency would result in heightened oxidative stress and inflammation, leading to increased susceptibility to PTB and prematurity-related morbidity and mortality in the offspring. Using Nrf2^−/−^ mice, we determined that Nrf2 plays a critical role in the gene-environment interactions that may determine susceptibility to PTB, and Nrf2 may be a potential target to reduce the risk of PTB and/or mitigate complications associated with PTB.

## Results

### Nrf2-deficient mice have increased susceptibility to preterm birth

**Figure 1:**
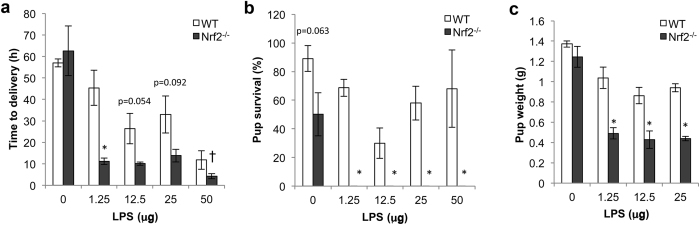
Nrf2-deficient mice have increased susceptibility to adverse birth outcomes. WT and Nrf2^−/−^ dams were injected with intrauterine LPS at E17, and mice were assessed for time to delivery (**a**), rate of pup survival at birth (**b**), and birth weight (**c**). N = 6, 5 (0 μg), N = 10, 5 (1.25 μg), N = 8, 7 (12.5 μg), N = 5, 5 (25 μg), N = 3, 2 (50 μg) litters for WT and Nrf2^−/−^ mice respectively. All data represent mean ± SEM. *p < 0.05 by two-tailed t-test. ^†^Time to labor. At this dose of LPS, Nrf2^−/−^ mice did not deliver pups despite prolonged and strong contractions, and were thus euthanized for humane reasons.

We used the established model of intrauterine LPS-induced PTB to determine whether Nrf2-deficiency alters susceptibility to PTB. Intrauterine injection of 50 μg LPS into pregnant WT mice at E17 induced delivery within 24 h and resulted in approximately 50% survival of pups (Fig. 1a,b). However, Nrf2^−/−^ mice were remarkably sensitive to this model of PTB. Following intrauterine delivery of 50 μg LPS, Nrf2^−/−^ mice showed rapid signs of labor, including vaginal bleeding within 15 min and strong contractions at approximately 4 h after injection (Fig. 1a). Despite these strong contractions, the Nrf2^−/−^ mice did not deliver their pups. The mice became extremely lethargic and were euthanized due to ethical considerations.

Therefore, to clarify the role of Nrf2 as a modifier of susceptibility to PTB, we assessed birth outcomes after multiple lower concentrations of LPS. In WT mice, we observed a dose response, in which 50 μg LPS induced delivery within 24 h, while 12.5–25 μg resulted in delivery beyond 24 h, and 1.25 μg LPS resulted in term delivery on embryonic day 19 (45.4 ± 8.1 h after LPS treatment). However, Nrf2^−/−^ mice did not demonstrate a dose response, and they consistently exhibited accelerated time to delivery (Fig. 1a) and reduced pup weight (Fig. 1c). Most profoundly, none of the Nrf2^−/−^ pups were alive at birth after treatment with the lowest dose of LPS (Fig. 1b). Notably, among the (phosphate buffered saline (PBS)-treated control mice, Nrf2^−/−^ mice delivered fewer pups (Supplementary Figure S1) and showed a trend toward reduced pup survival (Fig. 1b) compared to WT mice; although, PBS-treated Nrf2^−/−^ mice exhibited similar birth weights (Fig. 1c) and time to delivery as WT mice (Fig. 1a). This indicates that Nrf2 alters some birth outcomes in the absence of LPS, but these phenotypes were dramatically enhanced in the presence of LPS. These results indicate that Nrf2 is a strong modifier of PTB outcomes.

### Placentas from Nrf2-deficient mice have increased oxidative stress and inflammatory cytokines

**Figure 2:**
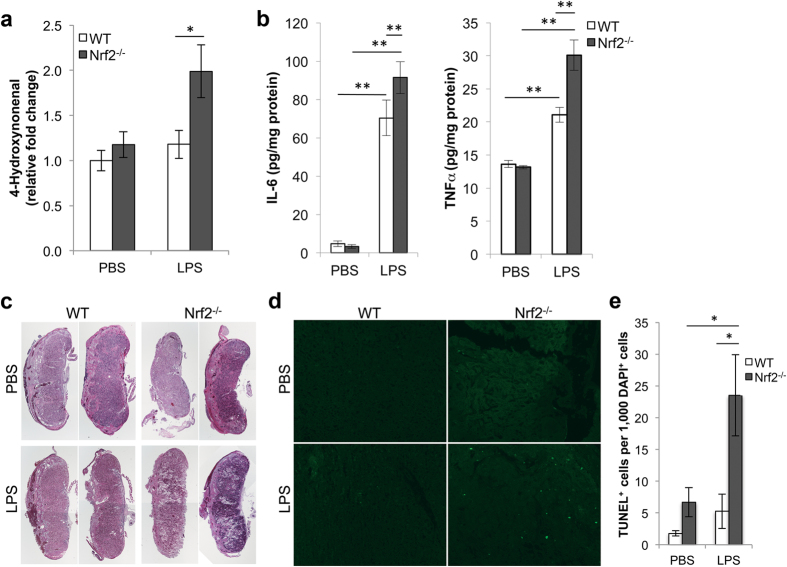
Placentas from Nrf2^−/−^ mice show increased levels of markers of oxidative stress, inflammation, and apoptosis after intrauterine injection of LPS. Pregnant mice were given an intrauterine injection of PBS or 25 μg LPS, and proximal placentas from the right uterine horn were harvested at 7 h after injection. (**a**) 4-HNE was quantified in placentas as a marker of oxidative stress. Values presented as fold increase compared to PBS-treated controls. N = 5–12 mice per group. (**b**) Levels of IL-6 and TNFα were quantified in placentas by ELISA. N = 4–7 mice per group. (**c**) Representative cross sectional images of proximal placentas stained with H&E. Each placenta was imaged at 20 × magnification and a composite of two 20 × images was generated using Photoshop. (**d**) Representative cross sectional images of proximal placentas stained with TUNEL. Each placenta was imaged at 100 × magnification. (**e**) Quantification of TUNEL-stained images. TUNEL + cells were normalized to the number of DAPI + cells per 100× field and stained cells were thresholded and quantified using the Nikon Elements software. N = 5–7 litters per group, with multiple placentas combined from each litter and treated as a single sample. All data represent mean ± SEM; *p < 0.05, **p < 0.01 as determined by two-way ANOVA followed by Tukey post-hoc test.

The placenta consists of both fetal and maternal tissue and plays a crucial role during fetal development. Oxidative stress and inflammation are key drivers of preterm delivery, and Nrf2 has a well-established role in reducing oxidative stress. Thus, we hypothesized that Nrf2^−/−^ mice display increased oxidative stress in response to LPS. Lipid peroxidation (4-hydroxynonenal [4-HNE]), a marker of oxidative damage, was quantified in placentas after intrauterine injection of 25 μg LPS or PBS at E17. Placentas were harvested at 7 h after LPS/PBS injection, shortly before the earliest signs of labor were observed in Nrf2^−/−^ mice. Placentas from Nrf2^−/−^ mice exhibited a significant increase in LPS-induced 4-HNE concentration compared to placentas from WT mice (Fig. 2a, p < 0.05 by two-way ANOVA). TNFα and IL-6 have been identified as important inflammatory determinants of susceptibility in this model of PTB. Among LPS-injected mice, both IL-6 and TNFα were significantly higher in Nrf2^−/−^ mice compared to WT mice (Fig. 2b, p < 0.01 by two-way ANOVA). Thus oxidative and inflammatory markers associated with PTB were exacerbated in Nrf2^−/−^ mice.

### Placentas from Nrf2-deficient mice have increased cell death

Histological sections of placentas from Nrf2^−/−^ mice treated with LPS revealed structural changes that were typified by increased empty spaces and disorganized orientation of the villi on the fetal side (Fig. 2c). Terminal deoxynucleotidyl transferase dUTP nick end labeling (TUNEL) staining showed significantly increased apoptosis in the LPS-treated Nrf2^−/−^ placentas compared to WT (Fig. 2d–e, p < 0.05 by two-way ANOVA), revealing further placental abnormalities following LPS treatment. Among PBS control samples, TUNEL staining was higher in Nrf2^−/−^ placentas, although this increase was not statistically significant by ANOVA. Among LPS-treated Nrf2^−/−^ mice, TUNEL+ cells were distributed throughout both fetal and maternal sides of the placenta (Supplementary Figure S2).

### Transcriptomic changes in placentas from WT and Nrf2^−/−^ mice

**Figure 3:**
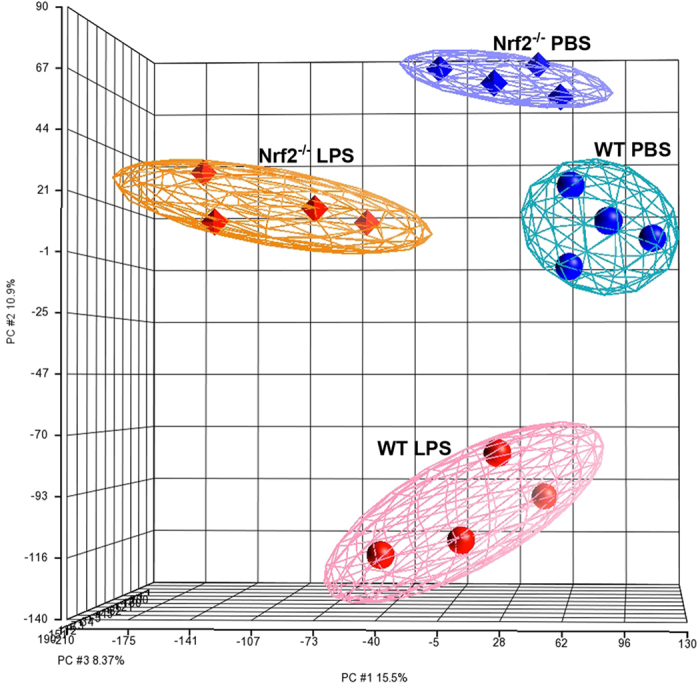
Principal component analysis of RNA expression from proximal placentas harvested at 7 h after intrauterine PBS or 25 μg LPS treatment.

**Figure 4:**
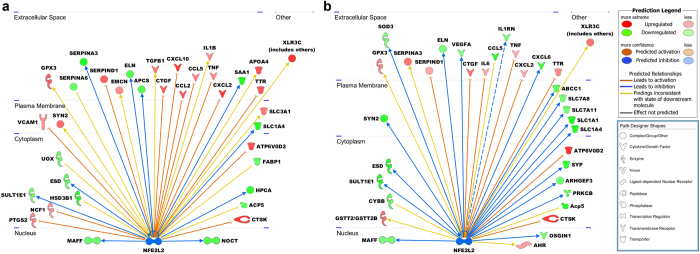
Ingenuity Pathway Analysis (IPA) of upstream regulators identified Nrf2 (NFE2L2) as a key regulatory network pathway that is inhibited in Nrf2^−/−^ PBS vs WT PBS (**a**) and Nrf2^−/−^ LPS vs WT LPS (**b**). Downstream gene symbols reflect increased (shades of red) or decreased (shades of green) expression in Nrf2^−/−^, as identified by microarray. Lines show predicted inhibition (blue) or activation (orange) of the downstream genes, according to IPA.

Nrf2 is a transcription factor that directly activates hundreds of target genes in response to a variety of environmental exposures; however, the transcriptional targets of Nrf2 in the placenta have not been investigated. Therefore, we performed a microarray analysis using RNA derived from placentas at 7 h after intrauterine LPS (25 μg) or PBS treatment. Principal component analysis of the microarray data showed that the samples clustered appropriately and clearly defined each group (Fig. 3). We then analyzed the transcription profiles using Ingenuity Pathway Analysis (IPA, Qiagen). IPA identified Nrf2 as a prominent upstream regulator based on annotated expression of downstream genes (z-score: −2.236 for PBS and −3.067 for LPS) (Fig. 4). Thus, the overall expression signatures reflect a strong decrease in Nrf2 signaling in the Nrf2^−/−^ mice.

### Pathway Analysis

We conducted functional pathway analysis using IPA. Using a differential expression threshold of greater than 2 SD fold change, IPA showed that, compared to WT PBS-treated mice, placentas from PBS-treated Nrf2^−/−^ mice showed activation of pathways related to inflammatory signaling, including signaling of pattern recognition receptors, chemokines, IL-8, IL-6, and NF-κB (Supplementary Table S1). Functional pathway analysis also showed increased production of nitric oxide and reactive oxygen species in Nrf2^−/−^ mice, primarily via increased expression of downstream pathway genes iNOS and NADPH oxidase subunits gp91-phox, p47-phox and p67-phox. Functional pathway analysis further revealed that among PBS-treated mice, Nrf2^−/−^ placentas showed increased inflammatory cell trafficking, chemotaxis and activation, as well as increased vascularization (Supplementary Table S2). Thus, among PBS controls, gene expression profiles in Nrf2^−/−^ mice were suggestive of elevated oxidative and inflammatory stress. However, these differences in gene expression under basal conditions were not manifested at the protein level (Fig. 2b). These data suggest that the gene expression profiles are more sensitive than protein markers of stress and indicate that Nrf2^−/−^ mice are primed for an exaggerated inflammatory response to LPS and increased oxidative stress.

**Figure 5:**
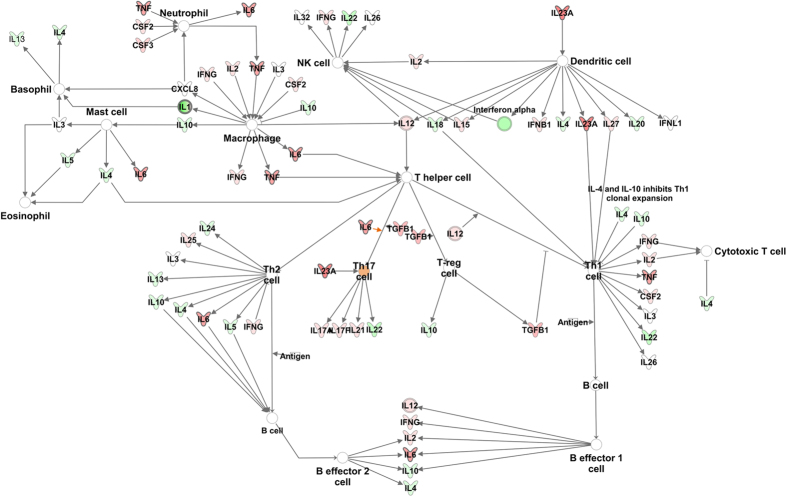
Ingenuity Pathway Analysis of placentas from LPS-treated mice showing the role of cytokines mediating communication between immune cells. Shades of red indicate relative up-regulation in Nrf2^−/−^ and green indicates down-regulation in Nrf2^−/−^. Orange indicates predictive activation.

Consistent with our findings in cytokine analysis (Fig. 2b), LPS induced expression of pro-inflammatory pathways in placentas from both WT and Nrf2^−/−^ mice. Gene expression of many of the pro-inflammatory cytokines, including IL-6 and TNFα, remained highest in the Nrf2^−/−^ LPS samples (Fig. 5), and functional pathway analysis identified increased myeloid cells. However, enrichment for canonical inflammatory signaling pathways was relatively weak when analyzed by IPA. Nrf2^−/−^ samples showed strong enrichment for cell cycle signaling, increased HMGB1 signaling, increased production of nitric oxide and reactive oxygen species, and inhibited RXR. Interestingly, functional pathway analysis also revealed increased autoimmune and T_H_17 responses in Nrf2^−/−^ mice in both the treatment groups. Collectively, the functional pathway analyses demonstrate that Nrf2 regulates numerous pathways that modify susceptibility to PTB.

## Discussion

Our present study demonstrated a direct role for Nrf2 in altering susceptibility to PTB. Nrf2 functions as a key activator of cytoprotective enzymes and antioxidants^20^,^21^. We have previously demonstrated that Nrf2^−/−^ mice have increased susceptibility to a variety of environmental diseases, including cigarette smoke-induced emphysema^32^, allergen-induced asthma^33^, and polymicrobial sepsis^34^. Thus, Nrf2 plays an important role in establishing gene-environment interactions that modify disease susceptibility. Since oxidative stress and inflammation are potent inducers of PTB, we hypothesized that Nrf2^−/−^ mice would exhibit elevated susceptibility to an established model of inflammation-induced PTB. Indeed, Nrf2^−/−^ mice were remarkably sensitive to this model, as indicated by significant decreases in time to delivery, birth weight, and pup survival. The birthweights were not matched for gestational age, and Nrf2^−/−^ mice often delivered many hours before WT mice, which may have partially accounted for the reduction in birthweight observed in Nrf2^−/−^ mice. However, the enhanced toxicity and mortality observed in Nrf2^−/−^ mice also contributed to this outcome. Thus, Nrf2 is required to ameliorate the adverse birth outcomes associated with intrauterine inflammation and oxidative stress.

Among PBS controls, Nrf2^−/−^ mice demonstrated some adverse outcomes compared to WT mice, including reduced litter size and a trend toward decreased survival of pups. These differences between WT and Nrf2^−/−^ mice may reflect a baseline difference in antioxidant capacity, which is consistent with findings from previous studies showing that Nrf2^−/−^ mice have subtle phenotypes under basal conditions, but are significantly more sensitive to inducers of oxidative stress and inflammation^22^,^23^. Additionally, the control mice underwent a laparotomy and PBS injection, which may have induced some background level of stress above that of a typical birth. Thus, under control conditions, Nrf2^−/−^ mice exhibited some adverse birth outcomes, but these adverse events were sharply increased in response to LPS. This conclusion is supported by our molecular endpoints, which showed differences in expression of pro-inflammatory mediators between placentas from PBS-treated WT and Nrf2^−/−^ mice but no significant differences in levels of cytokines or markers of oxidative stress or apoptosis. We did not observe a significant reduction in birthweight of PBS-treated Nrf2^−/−^ mice. However, Nrf2^−/−^ mice were recently demonstrated to exhibit intrauterine growth restriction^35^. In our current study, PBS-treated Nrf2^−/−^ mice exhibited a slight delay in delivery compared to WT controls that was not significant and a slight decrease in birthweight that was not significant. Thus, we could speculate that if the PBS-treated WT and Nrf2^−/−^ mice had been gestationally matched, these differences in birthweight may have been greater than what was observed and more consistent with the results published by Kweider *et al*.

Following intrauterine LPS treatment, we observed that placentas from Nrf2^−/−^ mice generated increased levels of 4-HNE, a marker of oxidative stress. Nrf2 regulates the inducible expression of numerous antioxidative genes, including superoxide dismutase (SOD), glutathione peroxidase, glutathione S-transferase (GST), and glutathione synthesis enzymes, and thus plays a vital role in reducing oxidative damage and inflammation due to various environmental stressors. Oxidative stress is a primary etiological factor in PTB. Data from previous studies show that, compared to term newborns, cord blood from preterm newborns contains significantly elevated levels of markers of oxidative stress, including increased lipid peroxidation, DNA adduct formation, and protein carbonylation, as well as decreased antioxidant capacity and vitamins A, C, and E^12^,^14^. Several of these oxidative stress markers have been shown to negatively correlate with birth weight and gestational age of the newborn^14^. Preterm mothers and neonates also have elevated levels of plasma nitric oxide and lipid peroxidation and decreased glutathione and glutathione peroxidase activity^15^,^16^,^17^,^36^. Antioxidants have been shown to reduce oxidative stress and inflammatory markers in both human gestational tissues and experimental models of PTB. In human gestational tissues (placenta, fetal membranes, and myometrium), treatment with antioxidants (curcumin, naringenin, or apigenin) reduced expression of LPS-induced IL-6, IL-8, and COX-2^37^. In animal models, numerous antioxidants have shown beneficial effects on PTB, fetal death, and intrauterine growth restriction^38^,^39^,^40^,^41^. Thus, oxidative stress strongly correlates with PTB and other adverse birth outcomes, and therapies that reduce oxidative stress could have beneficial effects in reducing the risks of PTB and its associated complications.

While antioxidant therapies may have some benefit in reducing the risk of PTB, activation of *Nrf2* is likely to be a more effective strategy in some women who are at high risk for PTB. Unlike antioxidant-based therapies that stoichometrically scavenge individual oxidants, *Nrf2* targets hundreds of genes to mount a coordinated and effective response. Previous studies from our laboratory and others have demonstrated that pharmacologic activation of Nrf2 has beneficial effects in models of emphysema^23^, chronic obstructive pulmonary disease (COPD) exacerbation^24^, viral infection^25^, asthma^26^, sepsis^27^,^28^, and radiation injury^29^. Similarly, 15-Deoxy-delta-12,14-prostaglandin J2 (15d-PGJ2), which is an activator of the Nrf2 pathway, was recently shown to suppress expression of thrombin-induced inflammatory mediators in human amnion mesenchymal cells, while intrauterine delivery of 15d-PGJ2 to pregnant mice significantly delayed thrombin-induced preterm delivery^42^. It is not clear whether this delay in PTB was directly due to activation of Nrf2, but it is consistent with findings in our current genetic study. Thus, there is tremendous therapeutic potential for activators of Nrf2, including among women who are at risk for PTB.

Previous studies have suggested that the Nrf2-dependent antioxidant pathway may play a role in PTB. For example, fetal membranes from preterm newborns with evidence of chorioamnionitis contain reduced Nrf2 expression compared to term and preterm membranes without chorioamnionitis, although the activity of Nrf2 remains unclear^43^. Diaphragms from preterm lambs contain reduced Nrf2 activity and reduced levels of antioxidants SOD2 and catalase^44^. This reduced pool of antioxidants makes preterm infants especially susceptible to the damaging effects of oxidative stress. Additionally, several genetic polymorphisms related to detoxification of oxidative stress have been associated with risk of PTB and related complications. Null genotypes in GST genes GSTM1 and GSTT1 and polymorphisms in SOD have been associated with low birth weight, reduced gestational age, and also correlate with elevated oxidative stress^17^,^45^. Polymorphisms in GSTM1, GSTM2, SOD1, SOD2, and catalase are more prevalent in infants with bronchopulmonary dysplasia, respiratory distress syndrome, retinopathy of prematurity, and intraventricular hemorrhage^46^,^47^. Furthermore, among women who smoked cigarettes during pregnancy (mean reduction in birth weight 377 ± 89 g), maternal GSTT1 genotype had a significant effect on birth weight reduction (285 ± 99 g [Present genotype] vs 642 ± 154 g [Absent genotype]), but no such association was observed among non-smoking pregnant women^10^. Thus, genetic determinants of oxidative stress have important roles in susceptibility to PTB as well as PTB-related complications through their interactions with environmental factors.

The pro-inflammatory transcription factors NF-κB and AP-1 are important activators of parturition^48^,^49^ and preterm delivery^50^, leading to the production of cytokines and prostaglandins that induce labor. Ingenuity Pathway Analysis identified higher baseline expression of pro-inflammatory pathways, including NF-κB, IL-6, and TNFα signaling pathways in Nrf2^−/−^ placentas, which remained elevated in response to LPS. Additionally, cytokine levels of IL-6 and TNFα were significantly elevated in Nrf2^−/−^ placentas after LPS treatment. Inhibitors of IL-6 and TNFα have both been shown to attenuate preterm delivery, fetal death, and intrauterine growth restriction in mice^51^,^52^,^53^. Interestingly, the transcriptional analysis also observed a significant increase in prostanglandin D2 synthase (Ptgds), which is a marker of preterm labor in women and promotes PTB in mice^54^. Pathway analysis also showed relative decreases in LXR/RXR activation in Nrf2^−/−^ PBS-treated placentas and inhibition of RXR function in Nrf2^−/−^ LPS-treated placentas. LXR/RXR may prevent parturition since it is antagonized by the labor-inducing prostaglandin F2α^55^ and suppresses NF-κB, Cox-2, and prostaglandin E2^56^,^57^. Thus, Nrf2^−/−^ placentas showed heightened expression of inflammatory and prostaglandin mediators that may promote labor.

Our studies have certain limitations. The mouse model that we used has a global deletion of Nrf2, and it would be difficult to dissect the contribution of this pathway in inflammatory cells, the placenta or uterus. Future studies on embryo transfer and the use of lineage-specific deletions would allow us to dissect the modifier role of Nrf2 on fetal and maternal tissues in PTB. These studies would also allow long-term follow up of the affected offspring, which is always an important consideration in studies concerning prematurity-related morbidity. Additionally, future studies are needed to identify the specific cell types in the placenta where Nrf2 is expressed, as well as elucidate how Nrf2 alters placental/fetal differentiation and vascularization throughout development.

In conclusion, Nrf2 is an important determinant of PTB. We have previously shown in a variety of disease models that pharmacologic activation of Nrf2 can reduce inflammation and oxidative stress, which are hallmark features of PTB. Additionally, prenatal treatment with sulforaphane, an Nrf2 activator^21^, showed beneficial effects in mouse models of postnatal hyperoxia^58^ and epidermolysis bullosa simplex^59^. Thus, Nrf2 activators, such as sulforaphane, may represent a promising strategy to reduce PTB. Future studies are needed to determine whether activation of the Nrf2-dependent response leads to improved birth outcomes in both experimental models and pregnant women.

## Methods

### Mice

We utilized WT and Nrf2^−/−^ mice on a CD-1 background, originally obtained from Dr. Masayuki Yamamoto, Tohoku University School of Medicine^20^, which were maintained in our animal facility as described previously^32^,^33^. Mice were mated overnight, and females were separated the following morning and checked for vaginal plugs. Mice containing vaginal plugs were considered to be at embryonic day 1 (E1). Mice were housed under controlled conditions for temperature and humidity, using a 12:12-h light-dark cycle. All experimental protocols were performed in accordance with the standards established by the US Animal Welfare Acts, as set forth in National Institutes of Health guidelines and in the Policy and Procedures Manual of the Johns Hopkins University Animal Care and Use Committee. All procedures were approved by the Johns Hopkins University Animal Care and Use Committee.

### Preterm Birth Model

We utilized a mouse model of intrauterine inflammation-induced PTB, which was validated by us and others^30^,^31^,^60^. Briefly, survival surgery was performed on timed pregnant mothers at E17, under general anesthesia with isoflurane and oxygen flow. After deep anesthesia was reached (respiration rate maintained at ~30 bpm), a laparotomy was performed. The abdomen was prepped with betadine and saline, and a vertical skin incision (avoiding mammary glands) was made. Sterility was maintained throughout the procedure. The right uterine horn was isolated and injected with 100 μl of LPS from *Escherichia coli*, 055:B5 in PBS or sterile PBS between the first and second fetus, not entering the amniotic cavity. Routine closure (3.0 vycril for peritoneal and staples for skin) was performed, and the dams were allowed to recover. Mice were video-recorded and watched carefully for signs of labor. For expression/biomarker measurements, placentas in the right uterine horn nearest the injection site were harvested at 7 h after injection, prior to delivery, which enabled standardization of time and proximity to injection.

### Cytokines and Oxidative Stress Markers

IL-6 and TNFα were measured by ELISA (eBioScience and R&D Systems, respectively). Lipid peroxidation was quantified as a marker of oxidative stress using the OxiSelect HNE Adduct Competitive ELISA Kit (Cell Biolabs, Inc.).

### Apoptosis

Placentas were harvested from WT and Nrf2^−/−^ mice at 7 h after intrauterine injection of PBS or 25 μg LPS. Placentas were fixed in 4% paraformaldehyde, then embedded in paraffin and sectioned at the midline. Sections were stained with TUNEL using the *in Situ* Cell Death Detection Kit, Fluorescein (Roche Diagnostics Corporation) and mounted using Fluoroshield with DAPI (Sigma-Aldrich). Slides were imaged at 100 × magnification using a Nikon Eclipse 80i equipped with a fluorescent camera and FITC filter. Images were quantified using Nikon Elements Imaging software, and the number of FITC-positive cells was normalized to the number of DAPI-positive cells.

### Microarray

RNA was isolated from placentas at 7 h after treatment with PBS or LPS (N = 4 mice per group) and processed for Affymetrix microarray analysis at the Boston University Microarray and Sequencing Resource. Raw Affymetrix MoGene_2.0 array CEL files were extracted and normalized by Robust Multiarray Averaging to produce log2 exon-level expression values using the Partek Genomics Suite v6.6 platform (Partek Inc. St Louis MO, USA). Exon-level data were then converted to gene-level using default mean summarization, and principal component analysis demonstrated clean separation of the four sample groups (Fig. 4). Between-class differential gene expression was evaluated using the one-way ANOVA model that provided for each comparison a fold change and the statistical significance of that fold change (p-value) for each transcript, 32,470 of which were associated with annotated genes. Examination of the log2 fold change of these gene transcripts showed the expected normal distribution for each group comparison, and their standard deviations from the mean was determined to establish fold change thresholds for further downstream functional analyses.

### Statistics

Individual comparisons were analyzed by unpaired Student’s two-tailed t-tests. Multiple comparisons were analyzed by two-way ANOVA with Tukey post-hoc tests. All measurements are presented as means ± standard error.

## Additional Information

**How to cite this article:** Sussan, T. E. *et al*. Nrf2 regulates gene-environment interactions in an animal model of intrauterine inflammation: Implications for preterm birth and prematurity. *Sci. Rep.* **7**, 40194; doi: 10.1038/srep40194 (2017).

**Publisher's note:** Springer Nature remains neutral with regard to jurisdictional claims in published maps and institutional affiliations.

## Supplementary Material

Supplementary Information
