# Supplementary material for: Nrf2 regulates gene-environment interactions in an animal model of intrauterine inflammation: Implications for preterm birth and prematurity
Source: Sci Rep. 2017 Jan 10;7:40194. doi: 10.1038/srep40194 (PMC5223218; doi:10.1038/srep40194)
Supplement: Supplementary Information [file srep40194-s1.doc]

**SUPPLEMENTARY INFORMATION**

**Nrf2 regulates gene-environment interactions in an animal model of intrauterine inflammation: Implications for preterm birth and prematurity**

Thomas E. Sussan, Kuladeep Sudini, C. Conover Talbot Jr, Xiaobin Wang, Marsha Wills-Karp, Irina Burd, Shyam Biswal

Supplementary Table S1. Canonical pathways differentially expressed in placentas according to Ingenuity Pathway Analysis (z-score >/<1).

|  | Ingenuity Canonical Pathways | z-score | Molecules |
| --- | --- | --- | --- |
| *Decrease in* | PPAR Signaling | -2.646 | FOS,PPARD,IL1B,PTGS2,NFKB2,TNF,TNFRSF11B |
| *Nrf2^-/-^ PBS* | Sperm Motility | -1.414 | PLA2G4E,PLA2G6,PLA2G4D,LCAT,PLCG2,PLA2R1,NPPC,Gucy1b2 |
|  | LXR/RXR Activation | -1.400 | APOA4,APOB,C4A/C4B,PON1,LYZ,LCAT,CCL2,SAA1,S100A8,GC,PON3, TNFRSF11B,AGT,HPX,TTR,APOM,AHSG,NFKB2,A1BG,APOA1,CD14, IL1B,PTGS2,TNF,RBP4 |
|  | Complement System | -1.342 | C4A/C4B,SERPING1,CD55,CFI,C1QB |
|  | Fc Epsilon RI Signaling | -1.342 | MAP2K6,PLA2G4E,PLA2G6,PLA2G4D,PLCG2,TNF |
|  | Apoptosis Signaling | -1.342 | CAPN6,PLCG2,NFKB2,TNF,FAS |
|  | PPARα/RXRα Activation | -1.134 | MAP2K6,APOA1,ACAA1,Cyp2c40 (includes others),TGFB1,PLCG2, Cyp2c70,TGFB2,IL1B,NFKB2 |
|  |  |  |  |
| *Increase in Nrf2^-/-^ PBS* | Production of Nitric Oxide and Reactive Oxygen Species in Macrophages | 3.300 | APOM,APOA4,APOB,NFKB2,FOS,PON1,NCF1,LYZ,RHOQ,APOA1, RHOD,PLCG2,HOXA10,S100A8,PPP2R2C,TNF,RBP4,TNFRSF11B |
|  | Dendritic Cell Maturation | 3.162 | ICAM1,HLA-A,FCGR2A,PLCG2,RELB,IL1B,HLA-DQB1,NFKB2,TNF, TNFRSF11B |
|  | HMGB1 Signaling | 3.051 | MAP2K6,FOS,VCAM1,RHOQ,ICAM1,CCL2,TGFB1,RHOD,TGFB2,IL1B,NFKB2, SERPINE1,TNF,TNFRSF11B |
|  | Role of Pattern Recognition Receptors in Recognition of Bacteria and Viruses | 2.828 | OAS1,TLR5,TGFB1,PLCG2,TGFB2,IL1B,C1QB,IFNA4,CCL5,NFKB2,TNF |
|  | Chemokine Signaling | 2.646 | CCR3,FOS,CCR5,CCL2,PLCG2,CCL5,CAMK2G |
|  | Renin-Angiotensin Signaling | 2.646 | FOS,CCL2,PLCG2,CCL5,NFKB2,TNF,AGT |
|  | IL-8 Signaling | 2.496 | PLD4,FOS,VCAM1,ANGPT2,RHOQ,GNG11,ICAM1,ANGPT1,RHOD,PTGS2,KDR,EIF4EBP1,PGF |
|  | LPS/IL-1 Mediated Inhibition of RXR Function | 2.236 | ALDH1B1,GAL3ST2,ALDH8A1,HMGCS2,HS6ST1,MAOB,SULT1E1, ALDH1A1,FABP6,ALDH1L2,CYP3A5,ALDH1A3,ALDH1A2,FABP4,CD14, IL1B,FABP1,FABP5,TNF,TNFRSF11B |
|  | Cholecystokinin/Gastrin-mediated Signaling | 2.121 | MAP2K6,FOS,RHOQ,RHOD,IL1B,EPHA4,PTGS2,TNF |
|  | Role of PI3K/AKT Signaling in the Pathogenesis of Influenza | 2.000 | CCR5,IFNA4,CCL5,NFKB2 |
|  | Pancreatic Adenocarcinoma Signaling | 2.000 | PLD4,TGFB1,TGFB2,PTGS2,NFKB2,PGF |
|  | IL-6 Signaling | 1.667 | MAP2K6,FOS,CRP,CD14,IL1B,HSPB7,NFKB2,A2M,TNF,TNFRSF11B |
|  | VDR/RXR Activation | 1.633 | CXCL10,WT1,SPP1,PPARD,TRPV6,HOXA10,TGFB2,CD14,IGFBP5, IGFBP1,CCL5 |
|  | TGF-β Signaling | 1.633 | MAP2K6,FOS,BMP4,TGFB1,BMP2,TGFB2,SERPINE1,INHBA |
|  | PKCθ Signaling in T Lymphocytes | 1.633 | CD3G,FOS,PLCG2,HLA-DQB1,NFKB2,CAMK2G |
|  | NF-κB Signaling | 1.604 | MAP2K6,TNIP1,NTRK2,BMP4,TLR5,PLCG2,BMP2,RELB,TDP2,IL1B,TNFAIP3, NFKB2,KDR,TNF,TNFRSF11B |
|  | Gαq Signaling | 1.414 | PLD4,RGS2,GNG11,RHOQ,RHOD,PLCG2,RGS4,NFKB2 |
|  | Ceramide Signaling | 1.342 | FOS,PPP2R2C,NFKB2,TNF,TNFRSF11B |
|  |  |  |  |
| *Decrease in* | Complement System | -2.236 | SERPING1,CD55,C3,C1S,CFI |
| *Nrf2^-/-^ LPS* | Cell Cycle: G2/M DNA Damage Checkpoint Regulation | -2.236 | TOP2A,CCNB2,PLK1,HIPK2,CDK1 |
|  | Regulation of Cellular Mechanics by Calpain Protease | -2.236 | CCNA2,RB1,CAPN6,CDK1,EGFR |
|  | eNOS Signaling | -1.667 | KNG1,ITPR2,Hspa1b,HSPA1A/HSPA1B,AQP1,SLC7A1,ITPR1,VEGFA, BDKRB2,HSPA8,CCNA2,AQP3,ITPR3,KDR,ESR1,PRKCB |
|  | p53 Signaling | -1.342 | RB1,WT1,HIPK2,GML,BIRC5,FAS,DRAM1 |
|  |  |  |  |
| *Increase in Nrf2^-/-^ LPS* | Mitotic Roles of Polo-Like Kinase | 2.236 | KIF23,PRC1,CCNB2,PLK1,CDK1,KIF11 |
|  | Estrogen-mediated S-phase Entry | 2.000 | CCNA2,RB1,ESR1,CDK1 |
|  | Type II Diabetes Mellitus Signaling | 1.890 | NGFR,IRS2,SMPD1,SOCS7,Irs3,TNF,TNFRSF11B,PRKCB |
|  | HMGB1 Signaling | 1.508 | MAP2K6,TLR4,RHOQ,AGER,RHOD,NGFR,TGFB2,RHOJ,IL6,RHOF,TNF, TNFRSF11B |
|  | Production of Nitric Oxide and Reactive Oxygen Species in Macrophages | 1.500 | APOB,RHOJ,ARG2,TLR4,ALB,RHOQ,RHOD,NGFR,HOXA10,CYBB,RHOF,TNF, RBP4,TNFRSF11B,APOD,PRKCB |
|  | Colorectal Cancer Metastasis Signaling | 1.460 | SMAD3,RHOJ,IL6,TCF7L1,BIRC5,TGFBR2,VEGFA,TLR4,CDH1,RHOQ,MMP23B,GNG11,RHOD,MMP8,TGFB2,MMP11,RHOF,MMP12,TNF,FZD2,MMP1,EGFR, WNT5A |
|  | Dendritic Cell Maturation | 1.414 | TLR4,IL36G,IL1RN,NGFR,TREM2,IL6,IL23A,TNF,TNFRSF11B |
|  | Cyclins and Cell Cycle Regulation | 1.342 | CCNA2,RB1,HDAC8,TGFB2,CCNB2,CDK1,HDAC5 |
|  | Integrin Signaling | 1.265 | CAPN6,RHOJ,ITGB8,ITGB7,ITGB3,TSPAN3,RHOQ,ACTA2,TSPAN1,RHOD, ITGA11,TSPAN4,RHOF,TSPAN6 |
|  | Glioma Invasiveness Signaling | 1.134 | RHOQ,RHOD,VTN,CD44,RHOJ,RHOF,ITGB3 |
|  | LPS/IL-1 Mediated Inhibition of RXR Function | 1.134 | FMO2,ABCG1,SOD3,GSTT2/GSTT2B,TLR4,HS6ST1,MAOB,IL36G,SULT1E1, FABP6,ALDH1A3,IL1RN,NGFR,ALDH1A2,FABP4,FABP5,ACOX3,TNF, TNFRSF11B,CYP2C8 |
|  | STAT3 Pathway | 1.134 | TGFBR2,NTRK2,NGFR,TGFBR3,SOCS7,KDR,EGFR |

Supplementary Table S2. Functional annotation according to Ingenuity Pathway Analysis (z-score >2).

|  | Diseases or Functions Annotation | Activation z-score |
| --- | --- | --- |
| *Increase in* | Accumulation of leukocytes | 4.034 |
| *Nrf2^-/-^ PBS* | Metabolism of reactive oxygen species | 3.982 |
|  | Inflammatory response | 3.831 |
|  | Accumulation of neutrophils | 3.482 |
|  | Cell movement of connective tissue cells | 3.477 |
|  | Vascularization | 3.446 |
|  | Lymphocyte migration | 3.434 |
|  | Activation of phagocytes | 3.331 |
|  | Accumulation of phagocytes | 3.329 |
|  | Accumulation of myeloid cells | 3.300 |
|  | Chemotaxis of granulocytes | 3.277 |
|  | Activation of antigen presenting cells | 3.276 |
|  | Growth of connective tissue | 3.268 |
|  | Formation of skin | 3.264 |
|  | Inflammation of respiratory system component | 3.206 |
|  | Activation of leukocytes | 3.201 |
|  | Cellular infiltration by macrophages | 3.166 |
|  | Hypersensitive reaction | 3.153 |
|  | Proliferation of connective tissue cells | 3.101 |
|  | Chemotaxis of connective tissue cells | 3.083 |
|  | Adhesion of vascular endothelial cells | 3.053 |
|  | Neovascularization | 2.996 |
|  | Cell movement of pericytes | 2.954 |
|  | Angiogenesis | 2.921 |
|  | Advanced malignant tumor | 2.900 |
|  | Metastasis | 2.900 |
|  | Arthritis | 2.859 |
|  | Arthropathy | 2.859 |
|  | Cell movement of natural killer cells | 2.794 |
|  | Cell movement of hepatic stellate cells | 2.779 |
|  | Adhesion of neutrophils | 2.757 |
|  | Cell movement of fibroblasts | 2.736 |
|  | Metabolism of hydrogen peroxide | 2.725 |
|  | Ion homeostasis of cells | 2.695 |
|  | Recruitment of neutrophils | 2.694 |
|  | Extravasation | 2.679 |
|  | Cytotoxicity of lymphocytes | 2.668 |
|  | Rheumatic disease | 2.648 |
|  | Metabolism of phospholipid | 2.622 |
|  | Migration of muscle cells | 2.613 |
|  | Activation of monocyte-derived dendritic cells | 2.609 |
|  | Cell movement of smooth muscle cells | 2.598 |
|  | Metabolism of membrane lipid derivative | 2.569 |
|  | Cytotoxicity of cells | 2.548 |
|  | Migration of connective tissue cells | 2.532 |
|  | T cell migration | 2.529 |
|  | Inflammation of lung | 2.523 |
|  | Synthesis of phospholipid | 2.443 |
|  | Cell death of antigen presenting cells | 2.426 |
|  | Migration of pericytes | 2.424 |
|  | Cytotoxicity of cytotoxic T cells | 2.422 |
|  | Synthesis of platelet activating factor | 2.414 |
|  | Development of body trunk | 2.399 |
|  | Proliferation of cells | 2.390 |
|  | Maturation of dendritic cells | 2.374 |
|  | Immediate hypersensitivity | 2.357 |
|  | Formation of osteoclasts | 2.351 |
|  | Cell movement of vascular smooth muscle cells | 2.334 |
|  | Fibrogenesis | 2.322 |
|  | Vascular lesion | 2.317 |
|  | Migration of hepatic stellate cells | 2.242 |
|  | NK cell migration | 2.241 |
|  | Excretion of protein | 2.219 |
|  | Concentration of cholesterol | 2.191 |
|  | Growth of malignant tumor | 2.173 |
|  | Quantity of blood cells | 2.168 |
|  | Differentiation of bone cells | 2.126 |
|  | Development of epithelial tissue | 2.034 |
|  |  |  |
| *Increase in* | Rheumatic disease | 2.557 |
| *Nrf2^-/-^ LPS* | Quantity of myeloid cells | 2.543 |
|  | Skin lesion | 2.523 |
|  | Tubulation of epithelial tissue | 2.361 |
|  | Progression of tumor | 2.348 |
|  | Arthropathy | 2.332 |
|  | Arthritis | 2.329 |
|  | Accumulation of granulocytes | 2.268 |
|  | Melanoma | 2.123 |
|  | Immediate hypersensitivity | 2.121 |
|  | Vascularization | 2.025 |
|  | Synthesis of DNA | 2.018 |
|  |  |  |

Supplementary Figure S1. Nrf2^-/-^ mice have decreased litter size. WT and Nrf2^-/-^ dams were injected with intrauterine PBS or LPS at E17, and number of pups was determined at either time of delivery or at dissection. N=6, 5 (0 μg), N=10, 5 (1.25 μg), N=8, 7 (12.5 μg), N=5, 5 (25 μg), N=3, 2 (50 μg) for WT and Nrf2^-/-^ mice respectively. All data represent mean ± SEM. *p<0.05 by two-tailed t-test.


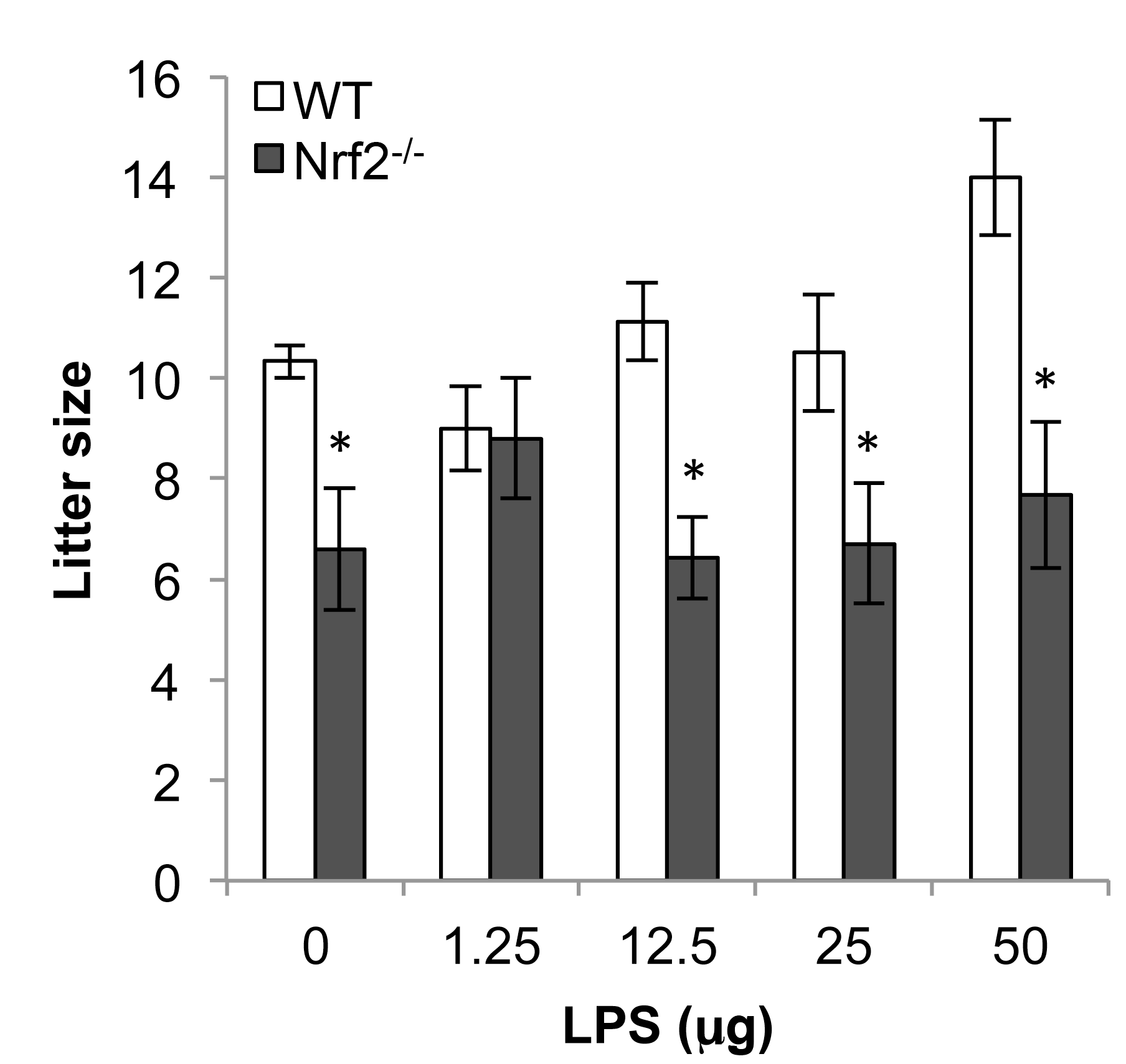


Supplementary Figure S2. Low magnification images of TUNEL-stained placentas from Figure 2D-E. Placentas from LPS-treated WT and Nrf2^-/-^ mice were imaged using a 4X objective lens, and images were assembled in Photoshop. Images demonstrate diffuse localization of TUNEL^+^ cells in both fetal and maternal sides of the placentas from Nrf2^-/-^ mice.


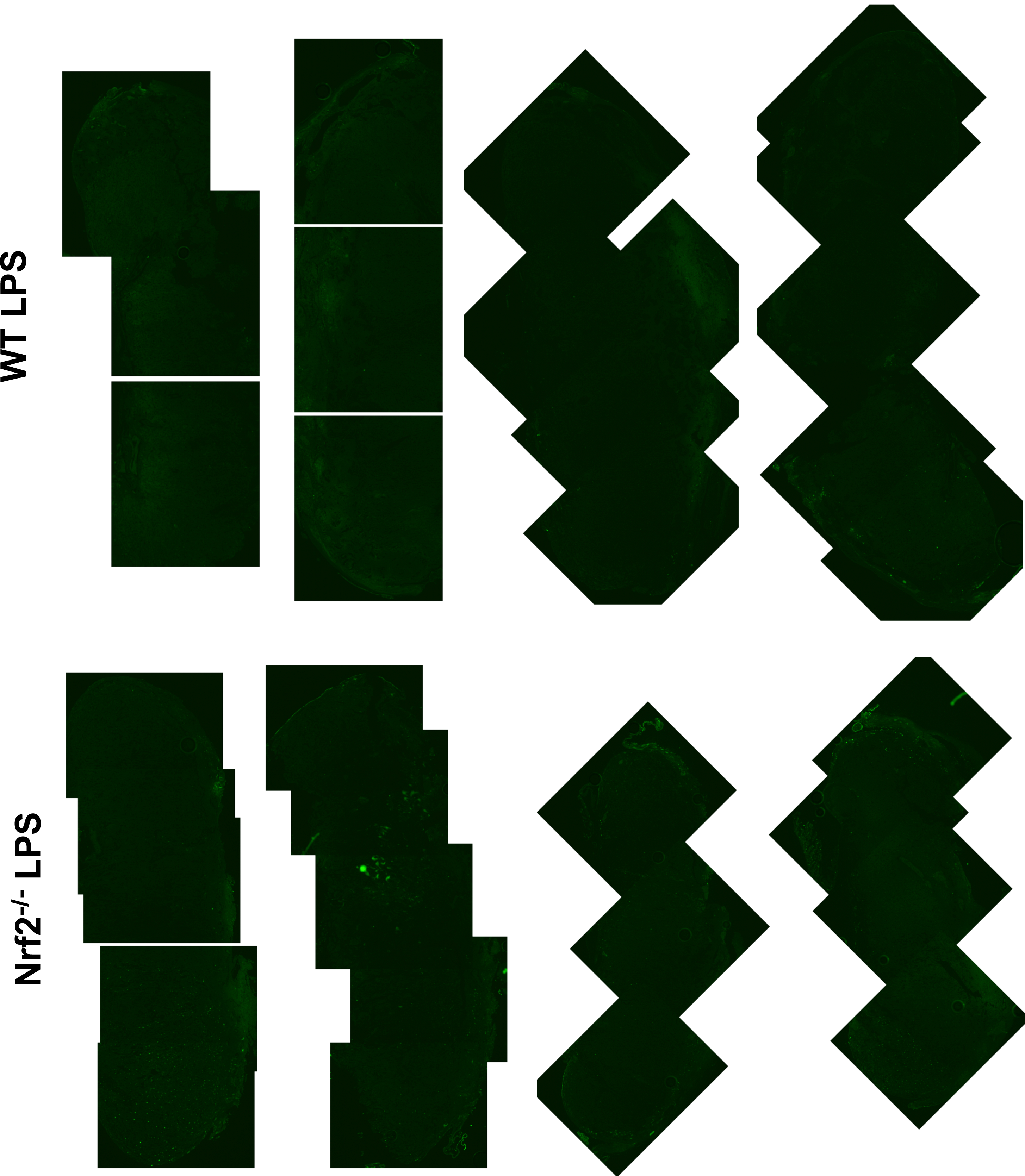
­­
